# The construction and validation of a clinical predictive model for somatic symptom disorders in epilepsy patients

**DOI:** 10.3389/fneur.2025.1758811

**Published:** 2026-01-12

**Authors:** Wenjing Shen, Changguo Zhang, Xuedan Pei, Zhongxia Shen, Xinhua Shen

**Affiliations:** 1 Department of Neurology, Huzhou Third People’s Hospital, Huzhou, Zhejiang, China; 2 School of Medicine, Huzhou University, Huzhou, Zhejiang, China; 3 Department of Nursing, Jifu Hospital, Xuzhou, Jiangsu, China; 4 Department of Psychosomatic Medicine, Huzhou Third People’s Hospital, Huzhou, Zhejiang, China

**Keywords:** comorbidities of epilepsy, model, predictive model, predictive modeling, somatic symptom disorder

## Abstract

**Objective:**

To investigate factors influencing somatic symptom disorder (SSD) in epilepsy patients and construct a cut-off point prediction model.

**Methods:**

Using structured interviews and based on DSM-5 diagnostic criteria, the 206 epilepsy patients included in this study were categorized into SSD and non-SSD (n-SSD) groups. Demographic and clinical data were collected, and assessments were conducted using the Quality of Life in Epilepsy (QOLIE-31), Generalized Anxiety Disorder-7 (GAD-7), Neuropsychiatric Disease and Disability Inventory-Extended (NDDI-E), and Pittsburgh Sleep Quality Index (PSQI). Age, negative life events, seizure anxiety, energy/fatigue, GAD-7, NDDI-E, and PSQI scores were identified as independent risk factors for SSD comorbidity in epilepsy. The constructed cut-off model demonstrated good predictive performance. External validation in an independent multicenter cohort is required prior to clinical implementation.

**Results:**

Compared with the n-SSD group, the SSD group exhibited statistically significant differences in age, age at onset, years of education, place of residence, number of comorbid physical illnesses, and adverse life events (all *p* < 0.05). The SSD group also showed significantly higher scores on GAD-7, NDDI-E, and PSQI, but lower total QOLIE-31 score and lower subscale scores for seizure worry, medication effects, energy/fatigue, life satisfaction, social functioning, and emotional well-being (all *p* < 0.05). Multivariate logistic regression analysis revealed that age (OR = 1.076, 95% CI: 1.015–1.141), negative life events(OR = 6.624, 95% CI: 2.130–20.606), seizure anxiety (OR = 0.945, 95% CI: 0.895–0.999), energy/fatigue (OR = 0.923, 95% CI: 0.872–0.977), and GAD-7 (OR = 1.274, 95% CI: 1.015–1.274) were independently associated with higher QOLIE-31 total scores. Fatigue (OR = 0.923, 95% CI: 0.872–0.977), GAD-7 (OR = 1.274, 95% CI: 1.037–1.565), NDDI-E (OR = 1.233, 95% CI: 1.038–1.442), and PSQI (OR = 1.375, 95% CI: 1.097–1.723) were independent predictors of SSD. The AUC of the nomogram model constructed based on the aforementioned factors was 0.939 (95% CI: 0.904–0.975), with an AUC of 0.907 following internal validation. The optimal risk probability cutoff value was 0.200 (based on the Yorden index), yielding a sensitivity of 84.7% and specificity of 95.3%. Calibration curve and decision curve analyses demonstrated good model calibration and clinical net benefit.

**Conclusion:**

Older age, exposure to negative life events, higher GAD-7, NDDI-E, and PSQI scores, and lower scores on the seizure worry and energy/fatigue dimensions of QOLIE-31 are independent risk factors for SSD in epilepsy patients. The constructed nomogram model demonstrates favorable predictive performance. External validation within an independent multicenter cohort is required prior to clinical implementation.

## Introduction

Epilepsy, as one of the most prevalent neurological disorders globally, has seen its co-occurrence of mental and psychological conditions become a key factor impacting patients’ quality of life and exacerbating familial and socioeconomic burdens. Previous studies have confirmed that the overall prevalence of mental disorders among people with epilepsy (PWE) reaches as high as 30 to 50% (1–3) with depression, anxiety disorders, and sleep disorders being relatively well-studied. However, systematic research on Somatic Symptom Disorder (SSD)—a condition characterized by persistent physical symptoms, excessive health concerns, and abnormal coping behaviors—remains comparatively scarce among PWE.

The diagnostic criteria for SSD underwent significant revision in the Diagnostic and Statistical Manual of Mental Disorders, Fifth Edition (DSM-5), addressing the shortcomings of DSM-IV’s overlapping classifications and ambiguous criteria for somatoform disorders. The new criteria place greater emphasis on symptom-related cognitive and behavioral abnormalities, enhancing the operational feasibility of clinical diagnosis. Current research indicates that the prevalence of SSD in the general population ranges from 4.5 to 7.4% (4, 5), while its incidence is significantly elevated among persons with epilepsy (PWE), with reported rates spanning 24.1 to 57.4% (6, 7). This suggests a potential specific associative mechanism between epilepsy and SSD. Existing research indicates that risk factors for SSD in PWEs span multiple dimensions: biologically, epilepsy-related structural and functional brain abnormalities (such as dysfunction in the frontal lobe-striatal circuit) may impair somatosensory processing, emotional regulation, and self-perception (8), providing a neural basis for SSD development; Psychologically, persistent anxiety about seizures, illness-related stigma (9), and maladaptive coping strategies may manifest psychological distress as somatic symptoms. Socially, external factors such as adverse life events and insufficient social support may exacerbate the severity and persistence of somatic symptoms.

It is noteworthy that SSD exhibits a high prevalence of comorbidity with anxiety, depression, and sleep disorders in people with epilepsy (PWE) (7) such co-morbidity not only complicates clinical diagnosis—often leading to underdiagnosis or misdiagnosis of SSD due to non-specific symptoms or overlap with epilepsy-related manifestations (e.g., headaches, fatigue)—but also markedly reduces treatment adherence, exacerbates functional impairment, and may trigger excessive healthcare-seeking behavior and resource wastage (10). Despite SSD’s significant impact on the prognosis of PWE, there is currently a lack of effective screening tools for assessing SSD risk in this population, and research on relevant predictive models remains largely unexplored. Consequently, this study aims to assess SSD comorbidity among epilepsy patients, explore the relationship between anxiety, depression, sleep disorders, and quality of life in this population by integrating demographic and clinical characteristics, and construct a nomogram model to predict the risk of SSD comorbidity in epilepsy patients.

## Objects and methods

### Object

This study was conducted between June 2023 and February 2024, recruiting adult epilepsy patients attending our hospital. Of the 218 patients initially approached, 12 were excluded upon review (due to missing key clinical data or incomplete questionnaires), resulting in a final cohort of 206 participants. This study was approved by the Ethics Committee of Huzhou Third People’s Hospital (Approval No. 2023-527), and all participants provided written informed consent. Inclusion criteria: (1) Diagnosis meeting the 2017 International League Against Epilepsy (ILAE) clinical diagnostic criteria for epilepsy (11), with a duration of one year or longer; (2) Age ≥ 18 years; (3) Being conscious, with fluent verbal expression, and able to cooperate with the structured interviews and complete all assessment scales for this study. Exclusion criteria: (1) Presence of severe chronic diseases; (2) Patients with significant cognitive impairment or psychiatric disorders rendering them unable to cooperate with the trial; (3) History of alcohol dependence or substance abuse; (4) Patients who had undergone any epilepsy-related surgical treatment. All patients underwent simultaneous structured interviews conducted by one attending neurologist and one attending psychiatrist. Referencing DSM-5 diagnostic criteria, patients were categorized into 43 cases in the SSD group and 163 cases in the non-SSD group (n-SSD group). This study was approved by the Ethics Committee of Huzhou Third People’s Hospital (Approval No. 2023-527). All participants voluntarily enrolled and provided written informed consent.

### Method

#### Collection of general clinical data

Collect general clinical data on patients, including gender, age, educational attainment, place of residence, marital status, presence of regular income, categories and types of other physical illnesses, and history of negative life events; alongside epilepsy-related variables such as age at onset, disease duration, seizure type (1 = generalized, 2 = focal, 3 = unclassified), presence/absence of epileptiform discharges on the most recent EEG, active epilepsy status (12, 13) (defined as ≥2 seizures within one year), and the names and quantities of antiepileptic drugs.

#### Evaluation of relevant scales

All personnel involved in the study underwent training in conducting interviews, administering relevant measurement tools, and selecting appropriate times to interview patients. Before each interview, participants were informed of the study’s objectives, required duration, and procedures. Scales were administered on-site, with completed questionnaires collected and immediately checked for quality. Any omissions or other issues affecting questionnaire quality were promptly addressed through supplementation or modification. (1) Quality of Life in Epilepsy Scale (QOLIE-31): The QOLIE-31 comprises seven subscales—seizure-related distress, life satisfaction, affect, energy/fatigue, cognitive function, medication impact, and social functioning—totaling 31 items. Higher total scores indicate better quality of life for patients with epilepsy. It demonstrates good reliability and validity among Chinese adults with epilepsy (14). (2) Generalized Anxiety Disorder Scale (GAD-7): This scale assesses distress caused by anxiety symptoms over the preceding 2 weeks. It comprises seven self-report items; a total score exceeding 10 indicates an anxiety disorder. Used for screening generalized anxiety disorder and evaluating symptom severity, it demonstrates good reliability and validity (15). (3) Epilepsy Depression Scale (NDDI-E): The NDDI-E comprises six self-report items, with a total score >12 indicating depressive status (16). This scale is straightforward to comprehend and complete, currently being the most prevalent tool for screening depression within the epilepsy field. (4) Pittsburgh Sleep Quality Index (PSQI): The PSQI assesses sleep habits over the preceding month through 24 items, comprising five observer-rated and 19 self-rated items. A score exceeding 7 indicates sleep disturbance (17). (5) Somatic Symptom Disorder - B Diagnostic Scale (SSD-12): The SSD-12 comprises 12 self-report items covering three dimensions: cognitive, affective, and behavioral. It corresponds to Diagnostic Criterion B for SSD in DSM-5. A total score ≥13 indicates a tendency toward SSD diagnosis (18). This measure assesses the psychological distress associated with somatic symptoms and demonstrates good reliability and validity in the population.

#### Statistical methods

All data analyses were performed using SPSS 26.0 software and R 4.3.1. Categorical variables were presented as frequencies and percentages and analyzed using Fisher’s exact test and the χ^2^ test. Before statistical analysis of continuous variables, data normality was assessed using the Shapiro–Wilk test; non-normally distributed continuous data were presented as median (M) and interquartile range (IQR), and the Mann–Whitney U test was used to evaluate differences between groups. Predictive model construction and validation: (1) Variable screening: Incorporate variables with *p* < 0.05 from univariate analysis into LASSO regression, employing 10-fold cross-validation and the lambda.min criterion for feature selection. (2) Model construction: Conduct multivariate logistic regression analysis on variables selected by LASSO, determining the final model using stepwise backward selection based on the Akaike Information Criterion (AIC). (3) Nomogram Construction: Based on the final model. (4) Performance Evaluation and Internal Validation: Calculate AUC, sensitivity, specificity, positive predictive value (PPV), negative predictive value (NPV), and their 95% confidence intervals. Determine optimal cut-off values using the Youden index. Plot calibration curves and perform Hosmer-Lemeshow tests. Assess clinical utility using decision curve analysis (DCA). Conduct internal validation via Bootstrap sampling (1,000 iterations). (5) Incremental value analysis: To evaluate the comprehensive model’s advantage, construct two simplified models for comparison: Model A (age and negative life events only); Model B (GAD-7, NDDI-E, and PSQI scores only). Compare AUC differences using the DeLong test.

## Results

### Comparison of clinical data between the SSD group and n-SSD group

Compared with the n-SSD group, the SSD group showed no statistically significant differences in male gender, marital status, fixed income status, disease duration, seizure type, presence of discharges on the last EEG, seizure frequency, or number of antiepileptic drugs (all *p* > 0.05). Compared with the n-SSD group, the SSD group exhibited statistically significant differences in age, age at onset, years of education, place of residence, number of comorbid physical illnesses, and negative life events (all *p* < 0.05). See Table 1 for details.

**Table 1:** Comparison of clinical data between the SSD group and the n-SSD group.

Variable	SSD (*N* = 43)	n-SSD (*N* = 163)	χ^2^/z/t	*p*
Sex^a^			1.403	0.236
Male	22 (51.20)	67 (41.10)		
Female	21 (48.80)	96 (58.90)		
Age^b^	64.23 (8.74)	56.00 (15.00)	−4.644	<0.001
Age of onset^b^	55.98 (10.07)	46.00 (16.00)	−3.874	<0.001
Course of disease^b^	7.00 (6.00)	7.00 (8.00)	−0.748	0.455
Education^a^			6.205	0.013
Below middle school	42 (97.70)	135 (82.80)		
Middle school or above	1 (2.30)	28 (17.20)		
Habitation^a^			4.929	0.026
City	33 (76.70)	95 (58.30)		
Countryside	10 (23.30)	68 (41.70)		
Marriage^a^			0.691	0.406
Married	39 (90.70)	140 (85.90)		
Unmarried	4 (9.30)	23 (14.10)		
Regular income^a^			0.005	0.944
Yes	14 (32.60)	54 (33.10)		
No	29 (67.40)	109 (66.90)		
Physical illnesses^a^			12.246	0.015
0	26 (60.50)	127 (77.90)		
1	10 (23.30)	27 (16.60)		
2	5 (11.60)	6 (3.70)		
3	0 (0.00)	2 (1.20)		
4	2 (4.70)	0 (0.00)		
5	0 (0.00)	1 (0.60)		
Negative life event^a^			12.969	<0.001
Yes	26 (60.50)	50 (30.70)		
No	17 (39.50)	113 (69.30)		
Seizure type^a^			0.798	0.713
1	5 (11.60)	13 (8.00)		
2	34 (79.10)	135 (82.80)		
3	4 (9.30)	15 (9.20)		
Electroencephalogram^a^			1.163	0.281
Yes	8 (18.60)	20 (12.30)		
No	35 (81.40)	143 (87.70)		
Stable^a^			0.003	0.955
Yes	30 (69.80)	113 (69.30)		
No	13 (30.20)	50 (30.70)		
Frequency^a^			1.559	0.453
0	30 (69.80)	113 (69.30)		
1	8 (18.60)	39 (23.90)		
2	5 (11.60)	11 (6.70)		
Medication quantity^a^			2.226	0.468
1	25 (58.10)	82 (50.30)		
2	15 (34.90)	62 (38.00)		
3	2 (4.70)	17 (10.40)		
4	1 (2.30)	2 (1.20)		

^a^Chi-square test [frequency (percentage), *N* (%)]; ^b^Mann–Whitney U test [median (interquartile range), median (IQR)]. *p*-value: Combined *p*-value for comparisons using the Mann–Whitney U test; *p*-value < 0.05 was considered statistically significant.

### Comparison of scale scores between the SSD group and n-SSD group

Compared with the n-SSD group, the patients in the SSD group had significantly higher scores on the GAD-7, NDDI-E, and PSQI scales (all *p* < 0.05), while the total score of QOLIE-31 and the scores of worry about episodes, drug effects, energy/fatigue, life satisfaction, social function, and emotion were significantly lower (all *p* < 0.05). The difference in cognitive function scores on the QOLIE-31 between the two groups was not statistically significant (*p* = 0.435). See Table 2 for details.

**Table 2:** Comparison of scale scores between the SSD and n-SSD groups [*M* (IQR)].

Variable	SSD (*N* = 43)	n-SSD (*N* = 163)	χ^2^/z/t	*p*
GAD-7^a^	10.00 (5.00)	6.00 (3.00)	−4.794	<0.001
NDDI-E^a^	15.65 (3.72)	11.80 (5.00)	−5.517	<0.001
PSQI^a^	7.51 (3.05)	4.40 (3.00)	−6.252	<0.001
QOLIE-31^a^	54.08 (5.46)	58.68 (6.10)	−4.801	<0.001
Seizure anxiety^a^	45.00 (15.00)	50.00 (10.00)	−5.509	<0.001
Drug effects^a^	43.14 (11.60)	55.00 (15.00)	−4.668	<0.001
Social functioning^a^	53.95 (8.13)	55.00 (10.00)	−2.053	0.040
Life satisfaction^a^	50.00 (10.00)	55.00 (10.00)	−3.860	<0.001
Energy/fatigue^a^	50.00 (10.00)	60.00 (15.00)	−3.791	<0.001
Social functioning^a^	60.00 (20.00)	60.00 (15.00)	−0.780	0.435
Emotional well-being^a^	65.00 (15.00)	60.00 (15.00)	−3.011	0.003

^a^Mann–Whitney U test [median (interquartile range), median (IQR)]. *p*-value: Combined *p*-value for comparisons using the Mann–Whitney U test; *p*-values < 0.05 were considered statistically significant.

### Variable filtering

#### LASSO regression

**Figure 1:**
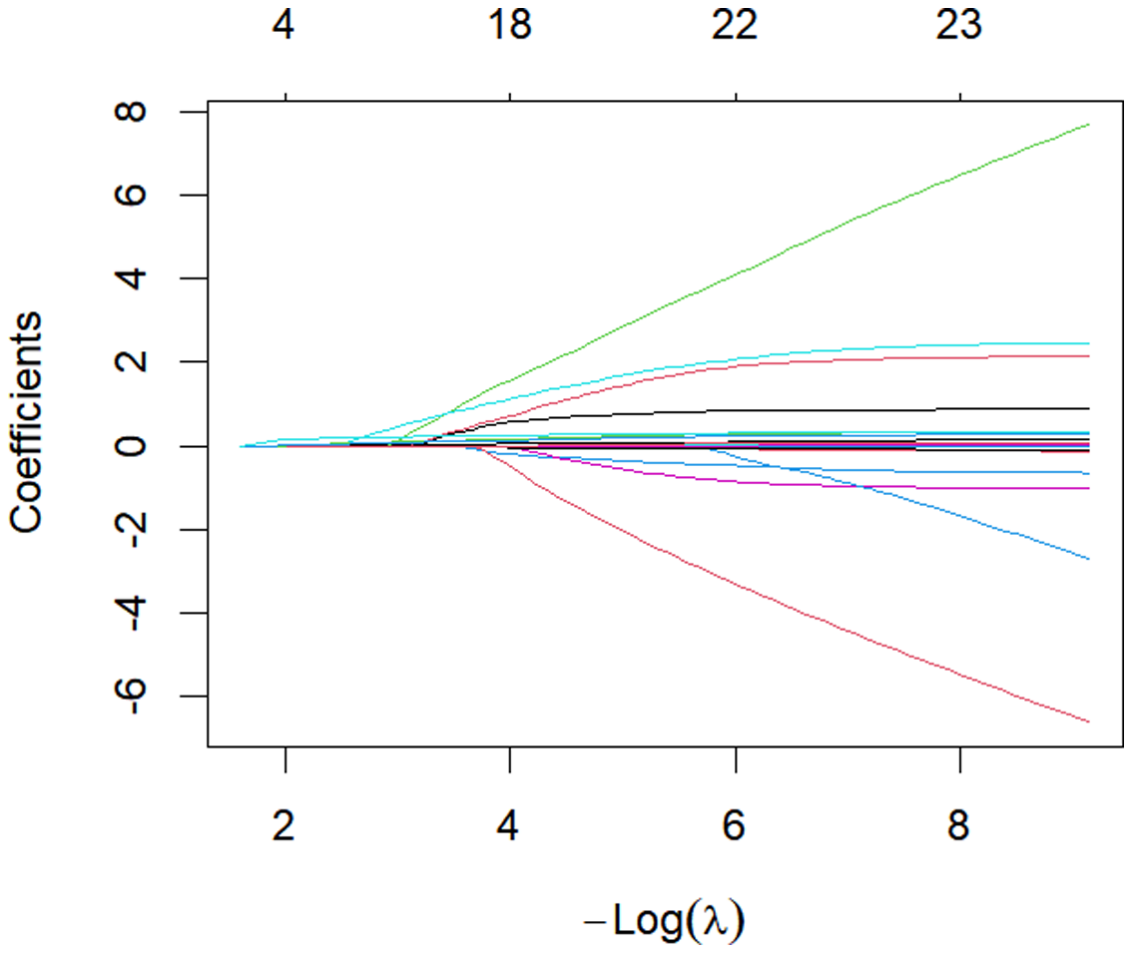
Cross-validation plot for the LASSO regression model. The optimal parameter (Lambda) in the LASSO model was determined using 10-fold cross-validation and the minimum standard. The vertical dashed line on the left indicates the minimum standard, while the vertical line on the right represents 1SE of the minimum standard (1-SE standard). Consequently, Lambda.min = 0.00703 was selected.

**Figure 2:**
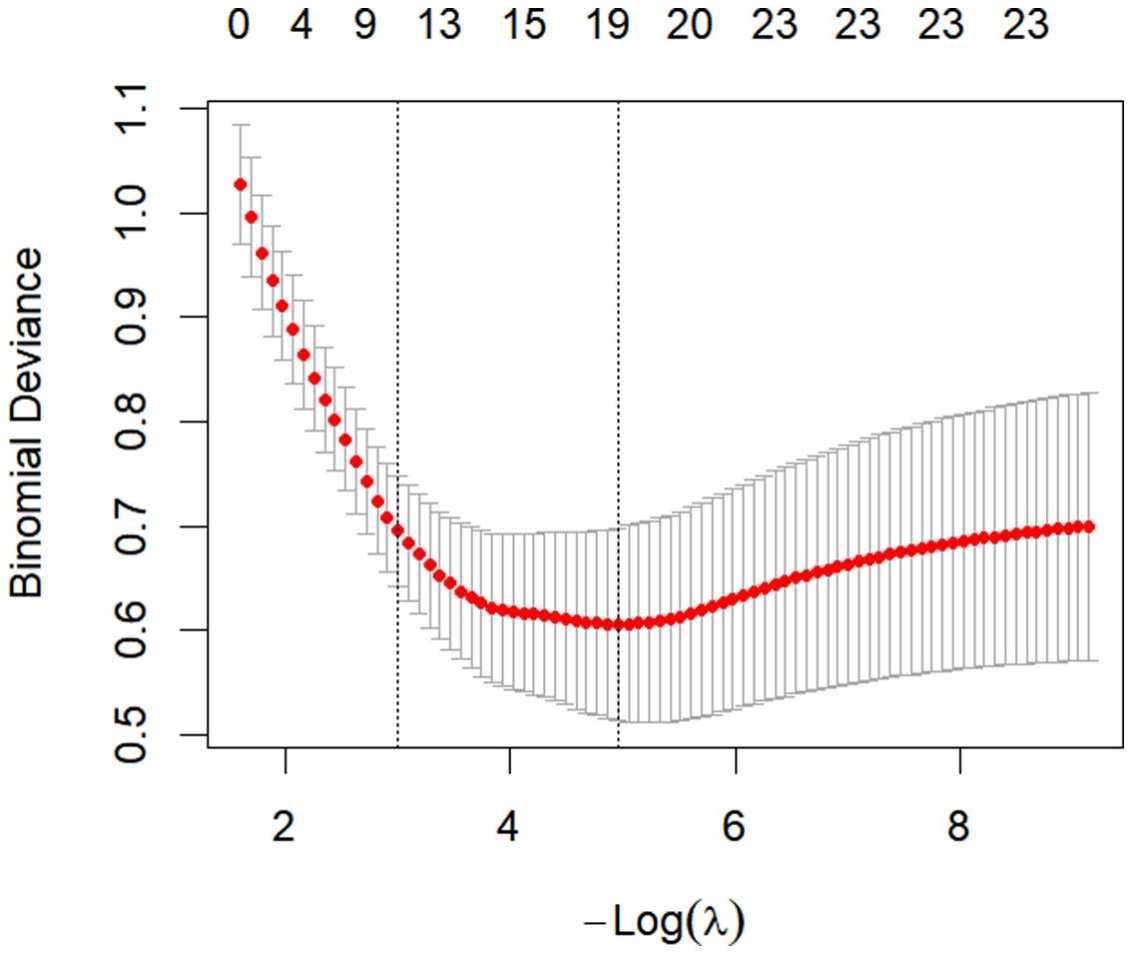
LASSO regression model coefficient plot. Distribution of LASSO coefficients for 10 factors. Vertical dashed lines at selected values were plotted using 10-fold cross-validation.

The 16 factors exhibiting statistically significant differences in Table 1 were employed as the initial model and selected via LASSO regression. Guided by the principles of maximum regularization and model stability, the optimal parameter *λ* was determined through 10-fold cross-validation using the minimum criterion. The 16 factors were subsequently reduced to 13, encompassing age, educational attainment, place of residence, number of physical illnesses, negative life events, GAD-7, NDDI-E, PSQI, QOLIE-31 total score, and concerns about recurrence, medication effects, energy/fatigue, and mood scores, all possessing non-zero coefficients (Figures 1, 2).

#### Univariate and multivariate logistic regression analysis

**Figure 3:**
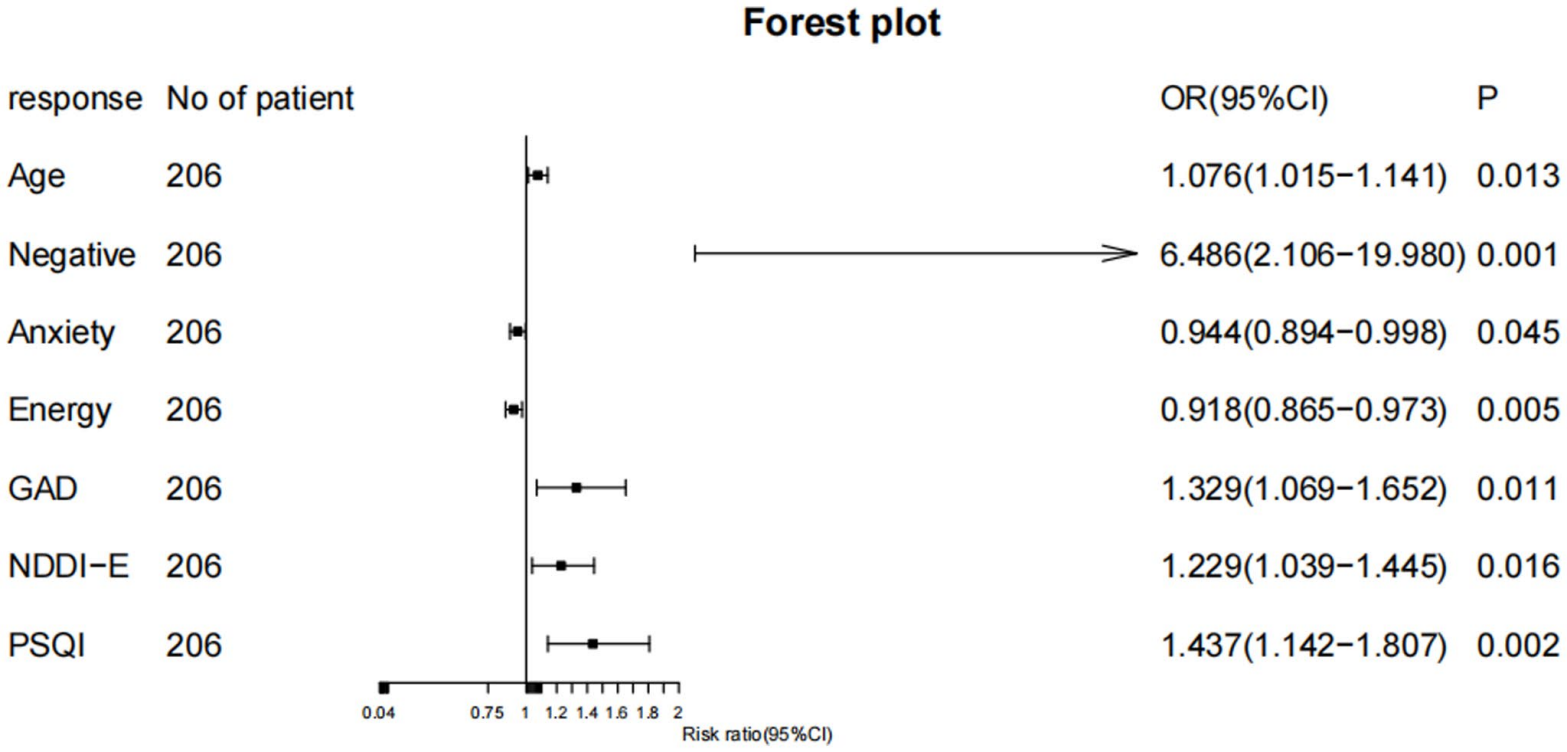
Forest plot of factors related to SSD.

Univariate analysis was conducted on the 13 variables evaluated by the Lasso regression model. Variables yielding *p* < 0.05 in univariate analysis (was this a subsequent statistical analysis? If so, please provide specific data) underwent multivariate logistic regression analysis using stepwise elimination to obtain an optimized model with the minimum AIC value. Results indicated that age (OR = 1.076, 95% CI: 1.015–1.141), negative life events (OR = 6.624, 95% CI: 2.130–20.606), seizure anxiety (OR = 0.945, 95% CI: 0.895–0.999), energy/fatigue (OR = 0.923, 95% CI: 0.872–0.977), GAD-7 (OR = 1.274, 95% CI: 1.037–1.565), NDDI-E (OR = 1.233, 95% CI: 1.038–1.442), PSQI (OR = 1.375, 95% CI: 1.097–1.723) were independent predictors of SSD (Figure 3). The AIC for the multivariate model was 116.1385. See Table 3 for details.

**Table 3:** Univariate and multivariate logistic regression analysis.

Variable	Univariate analysis			Multivariate analysis		
	OR	95%CI	*p*	OR	95%CI	*p*
Age	1.093	1.051 ~ 1.136	<0.001	1.076	1.015 ~ 1.141	0.013
Education	0.115	0.015 ~ 0.869	0.036			
Habitation	0.423	0.195 ~ 0.917	0.029			
physical illnesses	1.593	1.088 ~ 2.334	0.017			
Negative life events	3.359	1.676 ~ 6.731	0.001	6.486	2.106 ~ 19.980	0.001
QOLIE-31	0.828	0.768 ~ 0.894	<0.001			
Seizure anxiety	0.902	0.867 ~ 0.938	<0.001	0.945	0.895 ~ 0.999	0.044
Drug effects	0.925	0.894 ~ 0.956	<0.001			
Energy/Fatigue	0.938	0.905 ~ 0.973	0.001	0.923	0.872 ~ 0.977	0.006
Emotion	0.944	0.908 ~ 0.981	0.004			
GAD-7	1.490	1.282 ~ 1.732	<0.001	1.274	1.037 ~ 1.565	0.022
NDDI-E	1.327	1.195 ~ 1.474	<0.001	1.223	1.038 ~ 1.442	0.016
PSQI	1.742	1.445 ~ 2.100	<0.001	1.375	1.097 ~ 1.723	0.006

### Nomogram

**Figure 4:**
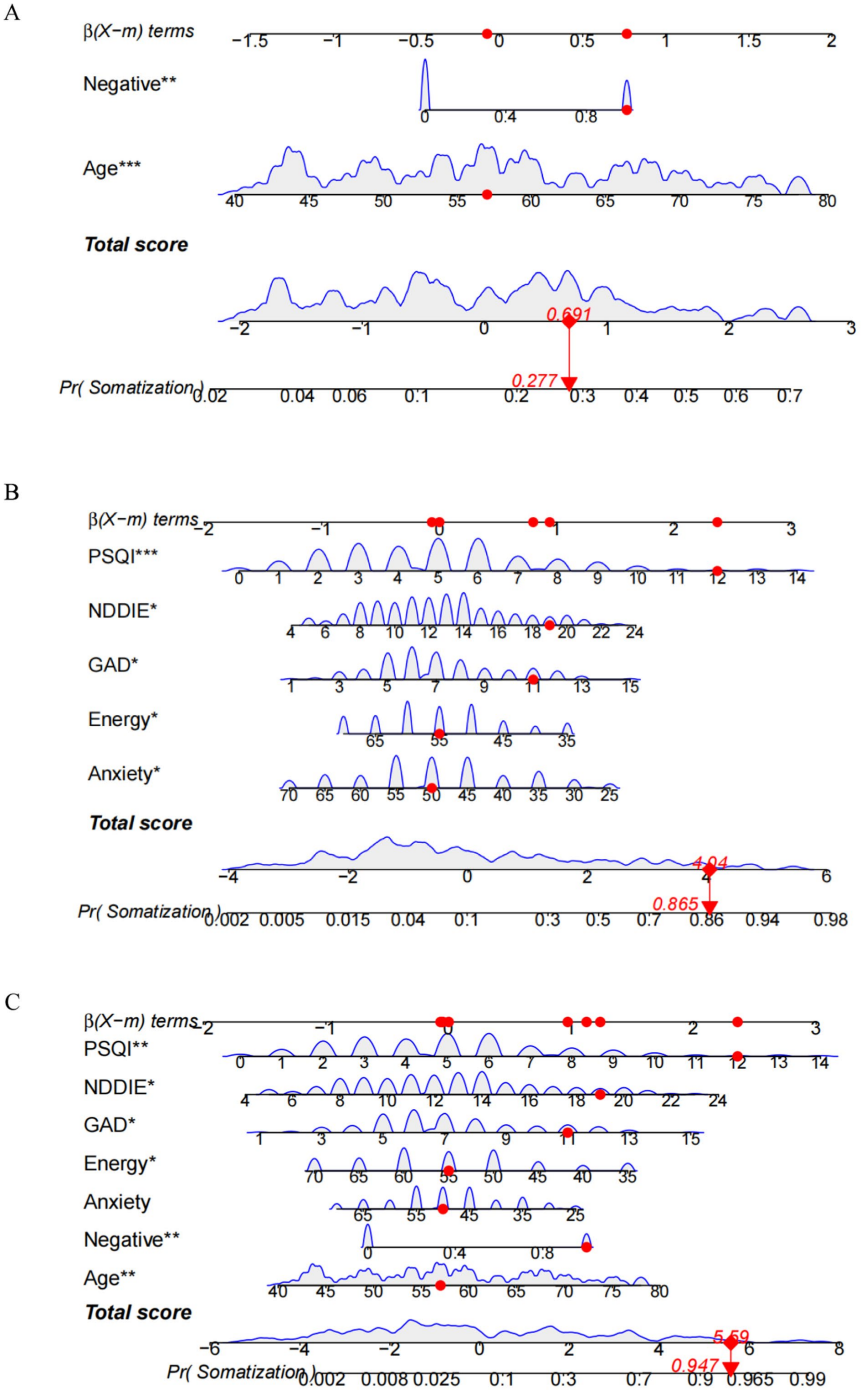
Nomogram models for somatic symptom disorder in patients with epilepsy. **(A)** Nomogram constructed with only demographic/clinical variables (age, adverse life events). **(B)** Nomogram constructed with psychological measurement scales (GAD-7, NDDI-E, PSQI, QOLIE-31 subscales). **(C)** Comprehensive nomogram for somatic symptom disorder in patients with epilepsy, including age, negative life events, QOLIE-31 seizure worry score, QOLIE-31 energy/fatigue score, total GAD-7 score, total NDDI-E score, and total PSQI score. Each of these variables was scored individually on a scale of 0 to 100. In addition, the scores of all variables were summed to generate a final total score, which corresponds to the probability of somatic symptom disorder in patients with epilepsy. Case verification: A 57-year-old patient with epilepsy had one negative life event, with a GAD-7 score of 11, NDDI-E score of 19, PSQI score of 12, energy/fatigue score of 50, and seizure worry score of 55. The probability of somatic symptom disorder was 27.7% when evaluated by Model A (including only age and negative life events), 86.5% by Model B (including only psychological measurement scales), and 94.7% by the comprehensive Model C.

Based on the aforementioned seven factors, bar charts were constructed (Figures 4A–C). Initially, a bar chart comprising solely demographic/clinical variables (age, adverse life events) was generated. Subsequently, bar charts incorporating psychometric scales (GAD-7, NDDI-E, PSQI, QOLIE-31 subscales) were developed. Finally, a comprehensive bar chart utilizing all seven variables was produced. Each of these variables was scored individually on a scale ranging from 0 to 100. Furthermore, the scores for all variables were summed to generate a final total score, which corresponds to the probability of epilepsy patients experiencing somatic symptom disorder.

#### Performance and internal validation of the nomogram

**Figure 5:**
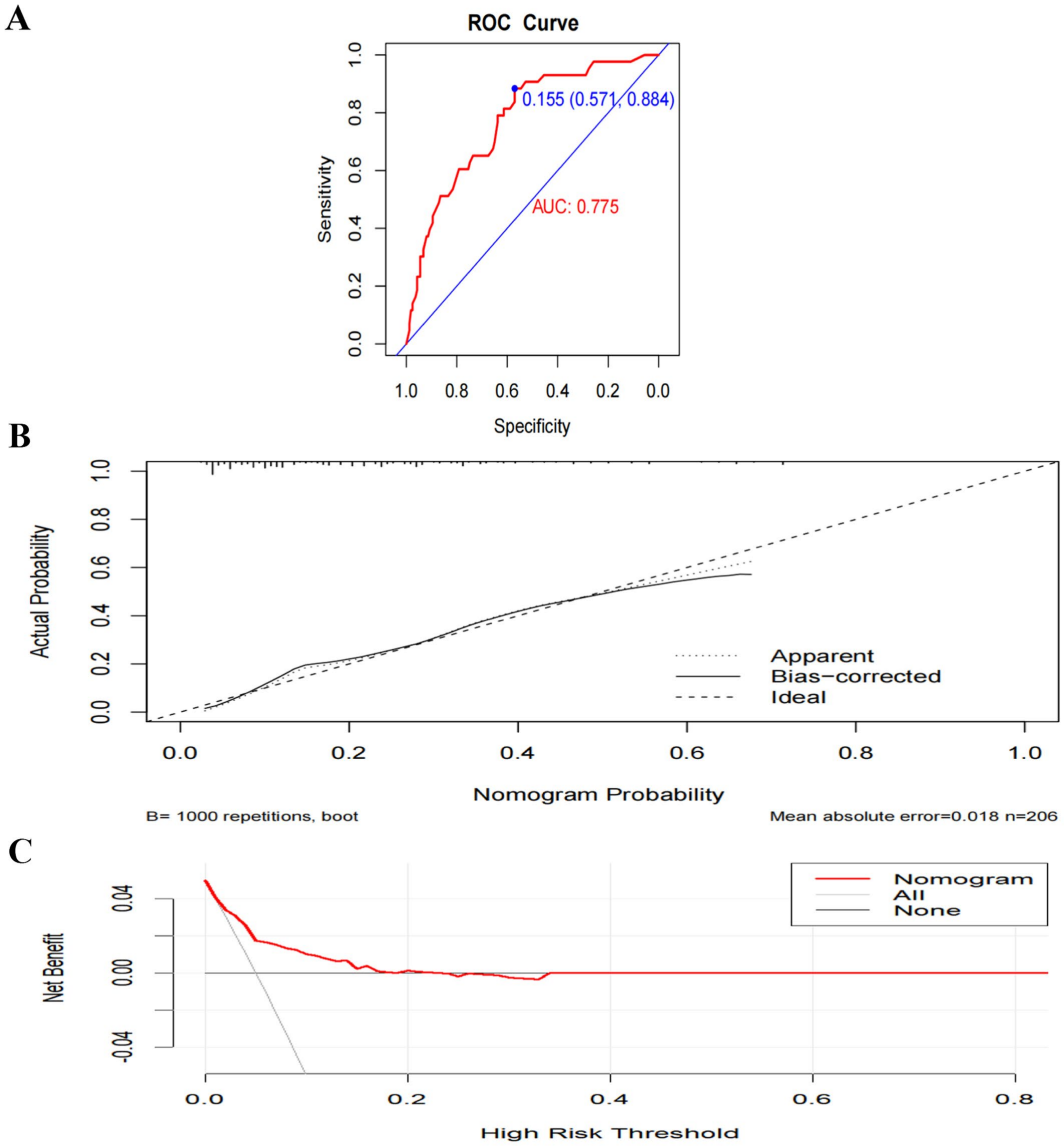
Performance and internal validation of Model A nomogram. **(A)** AUC curve; **(B)** Hosmer-Lemeshow test plot; **(C)** DCA curve.

**Figure 6:**
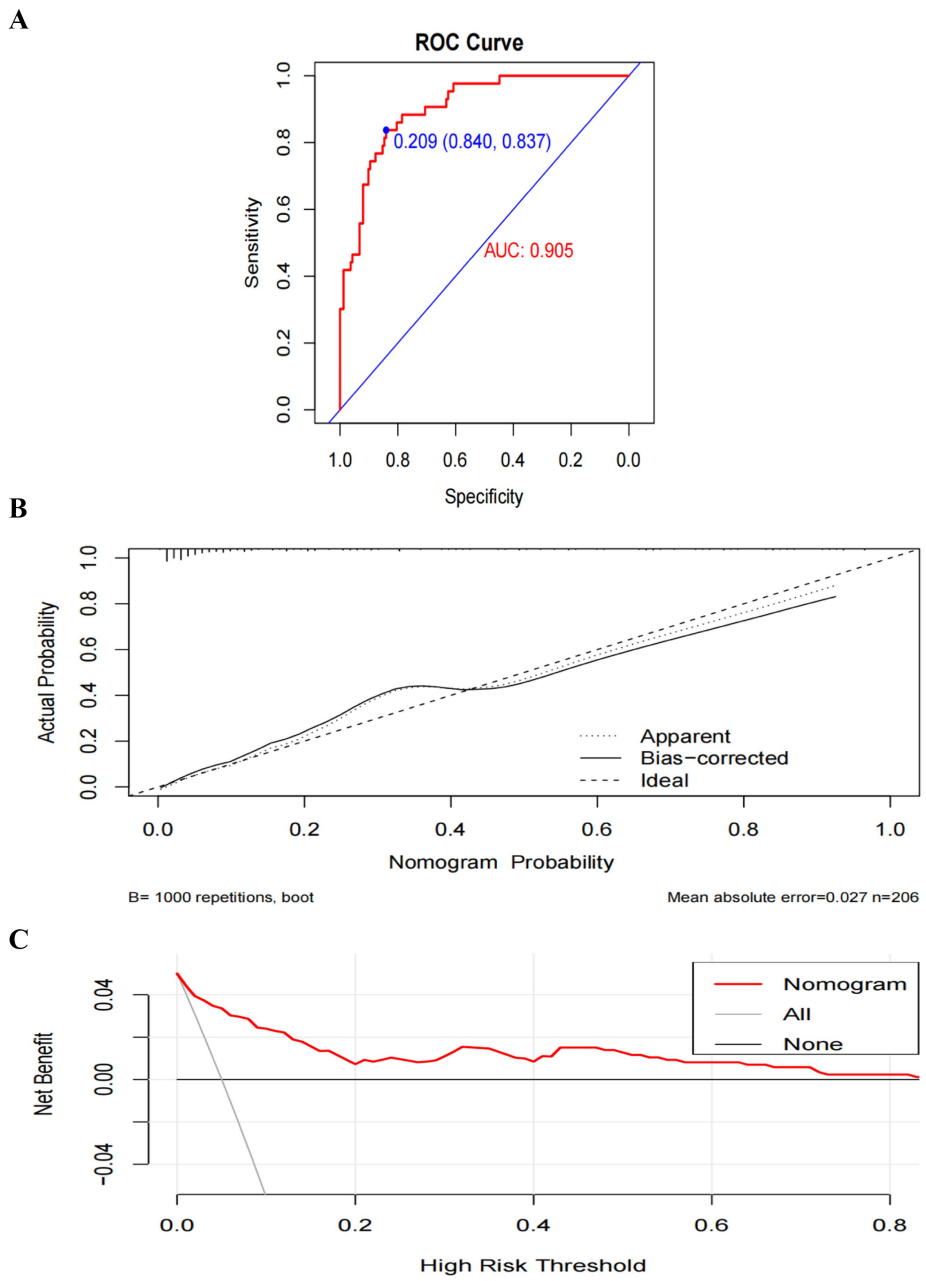
Performance and internal validation of Model B nomogram. **(A)** AUC curve; **(B)** Hosmer-Lemeshow test plot; **(C)** DCA curve.

**Figure 7:**
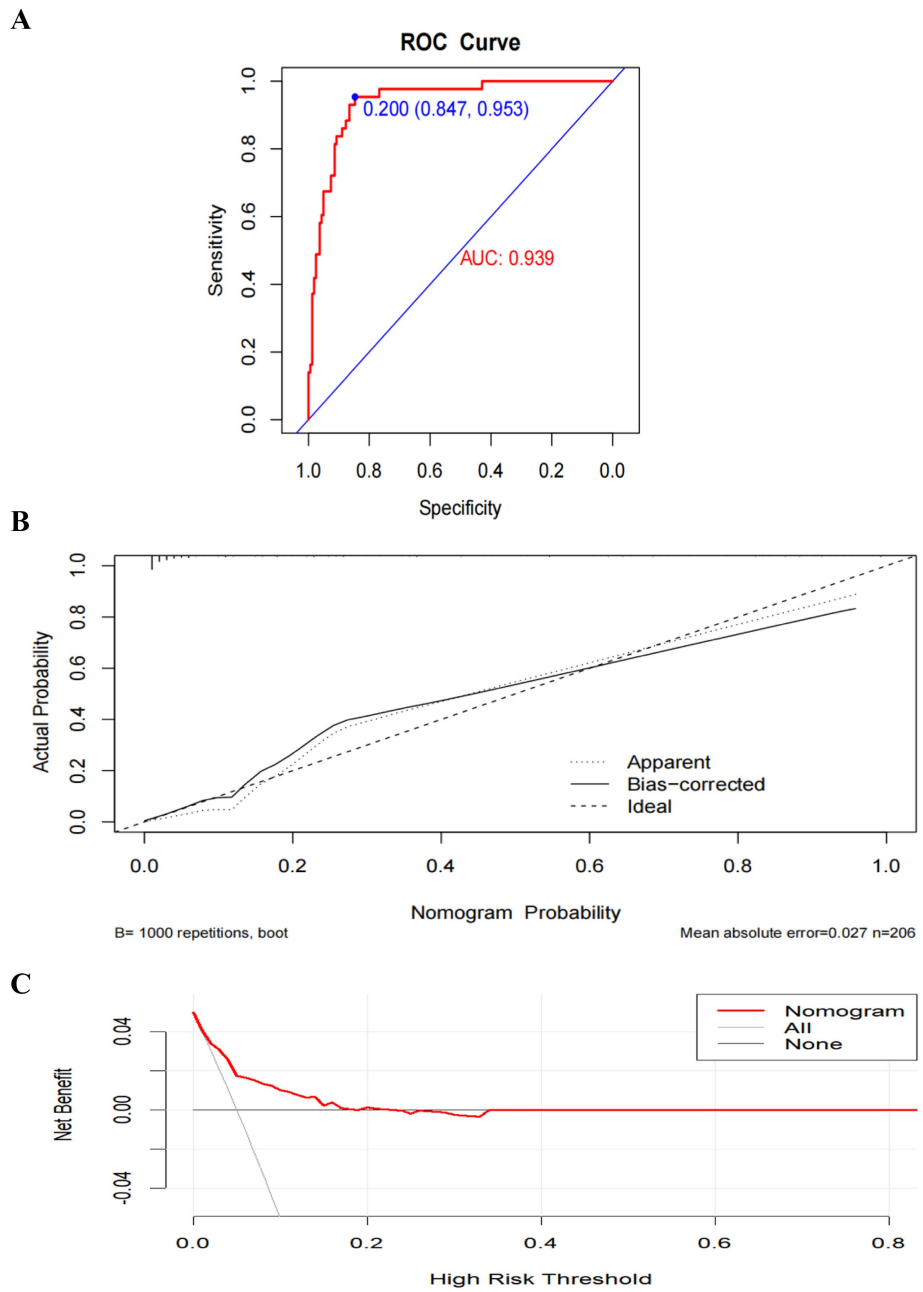
Performance and internal validation of Model C (comprehensive model) nomogram. **(A)** AUC curve; **(B)** Hosmer-Lemeshow test plot; **(C)** DCA curve.

Model A yielded an AUC of 0.775 (95% CI 0.699–0.851) (Figure 5A). The optimal risk probability cutoff determined by the Youden index was 0.155, yielding a sensitivity of 88.40% (95% CI: 74.90–96.10), specificity of 57.10% (95% CI: 49.00–64.90), positive predictive value (PPV) of 34.50%, and negative predictive value (NPV) of 94.90%. The Hosmer-Lemeshow test yielded χ^2^ = 8.923, *p* = 0.444 (Figure 5B). The decision curve analysis (DCA) curve illustrates the model’s clinical net benefit (Figure 5C). Model B demonstrated an AUC of 0.905 (95% CI: 0.860–0.950) (Figure 6A). The optimal risk cutoff determined by the Youden index was 0.209, yielding a sensitivity of 83.70% (95% CI: 69.30–93.20), specificity of 84.00% (95% CI: 77.50–89.30), positive predictive value (PPV) of 58.50%, and negative predictive value (NPV) of 94.80%. The Hosmer-Lemeshow test yielded χ^2^ = 3.288, *p* = 0.952 (Figure 6B). The decision curve analysis (DCA) curve illustrates the model’s clinical net benefit (Figure 6C). The model’s area under the curve (AUC) was 0.939 (95% CI: 0.904–0.975) (Figure 7A). The optimal risk probability cutoff determined by the Youden index was 0.200, yielding a sensitivity of 84.70% (95% CI: 71.40–93.20%), specificity of 95.30% (95% CI: 91.20–97.90%), with a positive predictive value (PPV) of 82.10% and a negative predictive value (NPV) of 96.30%. The calibration curve demonstrated good consistency (Hosmer-Lemeshow test, χ^2^ = 7.722, *p* = 0.562) (Figure 7B). Decision curve analysis indicated the model possessed clinical net benefit (Figure 7C). Following bootstrap internal validation, the area under the curve (AUC) was 0.907.

#### Model comparison and incremental value

To validate the superiority of the integrated model, we conducted an incremental analysis (Table 4). The predictive efficacy of the integrated model (AUC = 0.939) significantly outperformed Model A (AUC = 0.775, *p* < 0.001), which included only demographic/clinical factors, and Model B (AUC = 0.905, *p* = 0.027), which incorporated only psychological scale scores, demonstrating the necessity of integrating multidimensional information.

**Table 4:** Performance comparison of different predictive models.

Model	Predictor variables	AUC (95% CI)	Sensitivity (%)	Specificity (%)	PPV (%)	NPV (%)	DeLong test
Model A	Age, Negative life events	0.775 (0.699–0.851)	57.10	88.40	50.00	91.00	*p* < 0.001
Model B	Anxiety attacks, Energy/Fatigue, GAD-7, NDDI-E, PSQI	0.905 (0.860–0.950)	84.00	83.70	58.50	94.80	*p* = 0.027
Model C	Age, Negative life events, Anxiety attacks, Energy/Fatigue, GAD-7, NDDI-E, PSQI	0.939 (0.904–0.975)	84.70	95.30	82.10	96.30	For reference

## Discussion

Among the 206 epilepsy patients included in this study, the incidence of SSD was 20.9% (43/206), which is comparable to the 24.1% (150/631) reported by Zhou Dong et al. (7). This suggests a relatively high proportion of epilepsy patients co-occurring with SSD, yet clinical recognition rates remain low and the condition is insufficiently prioritized. This study found that patients in the SSD group were significantly older, potentially attributable to two factors: firstly, the study institution serves as a regional geriatric center, resulting in a higher proportion of elderly epilepsy patients and introducing selection bias; secondly, patients with late-onset epilepsy often present with more comorbidities and functional decline, potentially making them more likely to express psychological distress through somatic symptoms. Despite this age distribution bias, the inclusion of age as an independent predictor in the final model underscores the imperative for clinical practice to prioritize additional psychosomatic care for elderly epilepsy patients.

Through LASSO regression and multivariate logistic regression analysis, this study ultimately identified age, negative life events, concern about seizures (QOLIE-31 seizure concern dimension), energy/fatigue (QOLIE-31 energy/fatigue dimension), anxiety symptom severity (GAD-7), depression symptom severity (NDDI-E), and sleep quality (PSQI) as significant predictors. Fatigue (QOLIE-31 Energy/Fatigue dimension), anxiety symptom severity (GAD-7), depression symptom severity (NDDI-E), and sleep quality (PSQI) as seven independent predictors of SSD comorbidity in epilepsy. Older age, exposure to negative life events, higher scores on the GAD-7 and NDDI-E, elevated PSQI scores (indicating poor sleep quality), and lower scores on the seizure worry and energy/fatigue subscales of QOLIE-31 were significantly associated with an increased risk of SSD. Among these, advanced age was associated with an elevated risk of SSD onset, potentially linked to diminished physical functioning, greater prevalence of comorbid physical illnesses, and heightened preoccupation with health status among older adults. Furthermore, 36.9% (76/206) of patients had experienced adverse life events, with common types including bereavement of family members, adverse early childhood experiences, and sudden onset of physical illness, indicating the significant role of psychosocial factors in SSD development. Previous research (19) indicates that SSD arises from the combined effects of biological, psychological, and social factors. Multiple studies (8, 20) have confirmed structural and functional alterations in the brain of SSD patients, such as abnormalities in the frontal-striatal circuit. Concurrently, influenced by traditional culture, Chinese patients tend to express emotional distress through somatization (21). In this study, 23.3% of patients exhibited anxiety, 28.2% presented with depression, 25.2% experienced sleep disturbances, 42.2% had at least one co-occurring psychosomatic issue, and 86.0% of SSD patients co-occurred with at least one psychosomatic disorder. This indicates complex interactions between SSD and anxiety, depression, and sleep disturbances, which not only complicate clinical identification but may also exacerbate symptom severity, leading to repeated medical consultations (22). Clinical practice must prioritize differential diagnosis. Previous studies have suggested that seizure control, seizure severity, and antiepileptic drug use are closely associated with psychiatric comorbidity in patients (23). However, the present study found no statistically significant differences between SSD and non-SSD patients in terms of epilepsy duration, seizure type, presence of epileptiform discharges on the most recent electroencephalogram, seizure frequency, or number of AEDs used. This suggests that SSD and epilepsy may have distinct pathophysiological underpinnings, with its pathogenesis potentially emphasizing psychosocial factors rather than being solely driven by epilepsy itself. It should be noted that the structured interviews employed in this study may not fully distinguish whether patients also experienced psychogenic non-epileptic seizures; this issue warrants further clarification through long-term follow-up studies.

The regression model constructed in this study demonstrated excellent discriminatory capability within the training cohort (AUC = 0.939), with performance remaining robust following internal validation via Bootstrap sampling (corrected AUC = 0.907). Calibration curves and Hosmer-Lemeshow tests indicated good agreement between predicted and actual risks, while decision curve analysis revealed clinical net benefit across a broad range of thresholds. Notably, the ratio of SSD positive events (*n* = 43) to final predictor variables (*n* = 7) in this study was approximately 6.1, lower than conventional empirical recommendations. Consequently, model performance may be compromised in independent external cohorts, underscoring the imperative for external validation. Incremental analysis further confirmed that the predictive efficacy of this comprehensive model significantly outperformed simplified models incorporating only basic demographic/clinical variables (age, adverse life events) or relying solely on psychometric scale scores (GAD-7, NDDI-E, PSQI) (DeLong test *p*-values <0.05 in all cases). This demonstrates that integrating multidimensional information is essential for comprehensively assessing SSD risk.

In clinical practice, we recommend routinely collecting basic information such as age and history of adverse life events from all epilepsy patients attending consultations. We should proactively assess whether they experience persistent physical discomfort (e.g., dizziness, fatigue, palpitations) that cannot be fully explained by epilepsy or other somatic conditions. Standardized scales may be employed for rapid assessment: the GAD-7 for anxiety, the NDDI-E for depression, the PSQI for sleep quality, and the QOLIE-31 for quality of life (with particular emphasis on the dimensions of seizure-related worry and energy/fatigue). Should scale scores indicate abnormalities (GAD-7 > 10 points, NDDI-E > 12 points, PSQI > 7 points, or low scores in relevant QOLIE-31 dimensions), the individualized SSD risk probability may be further calculated using the nomogram developed in this study. Should the probability ≥ 0.200 (this cut-off point was determined based on maximizing the Youden index, yielding a sensitivity of 84.7% and specificity of 95.3%), it is recommended that a joint DSM-5 structured interview be conducted by neurologists and psychiatrists to confirm the diagnosis.

Clinical application examples can illustrate the tool’s usage intuitively. For instance, both presenting with recent onset of dizziness and palpitations, a 57-year-old male patient (Figure 4C) had recently experienced his father’s death. His GAD-7, NDDI-E, and PSQI scores were 11, 19, and 12 respectively, while his QOLIE-31 standardized scores for episode concern and energy/fatigue dimensions were 50 and 55. Calculating the corresponding scores for each variable using the nomogram and summing them yielded an SSD risk probability of approximately 94.7%. It is crucial to emphasize that before integrating this model and screening protocol into routine clinical practice, rigorous external validation across independent epilepsy cohorts from diverse geographical regions and medical centers is imperative to assess its real-world generalizability and clinical utility. For patients screening positive, integrated psychosomatic interventions should be promptly initiated. These should include psychological therapies such as cognitive behavioral therapy and mindfulness-based stress reduction, supplemented with pharmacological interventions where necessary. Concurrently, antiepileptic drug regimens should be optimized to minimize their potential impact on somatic symptoms.

In recent years, the integration of artificial intelligence in medical prediction models in recent years has expanded the methodological and clinical perspectives of our research. Contemporary AI‑assisted clinical prediction emphasizes not only performance but also model optimization, interpretability, and robust deployment. For instance, Li et al. (24) proposed a hyperparameter optimization approach, suggesting that systematic parameter tuning could enhance the accuracy and generalizability of our model. Yang et al. (25, 26) demonstrated the potential of attention‑based mechanisms and customized convolutional neural networks in multimodal electroencephalogram analysis and brain tumor grading, pointing toward future integration of time‑series EEG, neuroimaging, and other multimodal data in neurological prediction. Moreover, the use of ChatGPT in supporting neurodevelopmental disorders (27) illustrates the growing role of intelligent tools in neurological care. Nevertheless, as highlighted by Yee et al. (28) in cybersecurity research, AI implementation in clinical settings still encounters challenges related to robustness, trust, and secure system integration. Moving forward, the nomogram developed here may serve as a foundation for evolving into an intelligent, interpretable, and clinically embedded “hybrid prediction system” within electronic health records, enabling dynamic risk assessment and early warning for epilepsy‑related SSD.

The scatter plots constructed in this study provide a visualization tool for predicting SSD risk in epilepsy patients. Recent applications of artificial intelligence (AI) in epilepsy research have offered novel approaches to model optimization. For instance, the SeizureLSTM model proposed by He et al. employs attention mechanisms and optimal weighted feature integration for seizure detection, with its core advantage lying in efficient modeling of time-series data (24). This offers significant implications for expanding epilepsy-SSD prediction models: as the predictors in this study are predominantly cross-sectional data, future work could draw upon time-series modeling approaches. Integrating long-term follow-up data (such as changes in seizure frequency and dynamic trends in scale scores) could facilitate the construction of dynamic risk assessment models. This would further enhance the timeliness and accuracy of predictions, enabling the SSD prediction framework to be integrated into broader AI-assisted epilepsy management research systems.

This study retains certain limitations: the sample size was relatively small and the research was single-center, with an older age distribution among patients, which may impact the model’s generalizability. Furthermore, the study did not incorporate biological markers nor conduct long-term follow-up. We therefore emphasize that rigorous external validation across geographically diverse, multicenter cohorts of independent epilepsy patients is essential before considering any clinical application. Future work should involve large-scale, multicenter studies incorporating objective biological markers and longitudinal designs to further optimize the model’s applicability and predictive efficacy.

In summary, age, adverse life events, seizure anxiety, energy/fatigue, GAD-7, NDDI-E, and PSQI scores constitute key risk factors for SSD in epilepsy. The cut-off point model developed based on these factors demonstrates sound predictive performance and clinical utility, serving as an auxiliary screening tool for early identification of high-risk patients. Prior to routine clinical implementation, this model must undergo independent, multicenter external validation.

## Data Availability

The datasets presented in this article are not readily available because the dataset generated and analyzed during this study is not publicly available. This restriction arises from ethical guidelines and privacy protections for epilepsy patients. Requests to access the datasets should be directed to shenwenjingcxzc@163.com.
